# Postacute sequelae of SARS-CoV-2 (PASC) in nursing home residents: A case–control study

**DOI:** 10.1017/ash.2022.135

**Published:** 2022-05-16

**Authors:** Sophie Clark, Liza Marie Bautista, Karen Neeb, Ana Montoya, Kristen Gibson, Julia Mantey, Lillian Min, Lona Mody

## Abstract

**Background:** Postacute sequelae of SARS-CoV-2 (PASC) include fatigue, dyspnea, anxiety, and cognitive impairment. Few studies have explored the prevalence or presentation of PASC among nursing home (NH) residents. **Method:** A case–control study was conducted at 1 NH in Michigan in December 2021. Cases were defined as residents with SARS-CoV-2 infection between November 2, 2020, and October 8, 2021. Controls lived at the same NH during this interval and never tested positive for SARS CoV-2. Patient characteristics were compared between cases and controls using the Fisher exact test and Wilcoxon rank-sum test. Primary outcomes were functional decline, cognition, and adverse health outcomes. Outcomes were assessed by comparing measures on last observation to observations before COVID-19 diagnosis (cases) or to earliest observation (controls). Multivariable logistic regression assessed correlation between COVID-19 diagnosis and outcomes. **Results:** In total, 152 residents were identified for inclusion (147 included in final analyses, 76 cases, 71 controls); 5 were excluded due to insufficient data. We collected the following resident characteristics: 66% were aged ≥80 years; 73% were female; 95% were non-Hispanic white; 82% were long-stay residents; median of 3 comorbidities (IQR, 2–4). The mean number of follow-up observations was 2.60 (SD, 1.25). No significant differences in population characteristics were detected between cases and controls. Moreover, 106 patients (46 cases and 60 controls) had at least 1 follow-up visit and were thus included in the analyses to evaluate long-term outcomes. Among them, cases experienced significant declines in completing transfers (OR 5.65, p **Conclusions:** Nursing home residents with COVID-19 are more likely to enter hospice and have a higher mortality rate in the year following infection. Survivors experience significant functional decline in basic activities of daily living, specifically in the ability to transfer and dress. Larger studies are needed to further characterize our findings and to design interventions that can help overcome these long-term sequelae from COVID-19.

**Funding:** None

**Disclosures:** None

